# Clinical study on the prediction of ALN metastasis based on intratumoral and peritumoral DCE-MRI radiomics and clinico-radiological characteristics in breast cancer

**DOI:** 10.3389/fonc.2024.1357145

**Published:** 2024-03-19

**Authors:** Yunxia Wang, Yiyan Shang, Yaxin Guo, Menglu Hai, Yang Gao, Qingxia Wu, Shunian Li, Jun Liao, Xiaojuan Sun, Yaping Wu, Meiyun Wang, Hongna Tan

**Affiliations:** ^1^ Department of Radiology, People’s Hospital of Henan University, Zhengzhou, Henan, China; ^2^ Department of Radiology, Henan Provincial People’s Hospital, Zhengzhou, Henan, China; ^3^ Department of Radiology, People’s Hospital of Zhengzhou University, Zhengzhou, Henan, China; ^4^ Department of Radiology, Affiliated Cancer Hospital of Zhengzhou University &Henan Provincial Cancer Hospital, Zhengzhou, China; ^5^ Heart Center, People’s Hospital of Zhengzhou University & Henan Provincial People’s Hospital, Zhengzhou, China; ^6^ Beijing United Imaging Research Institute of Intelligent Imaging & United Imaging Intelligence Co., Ltd., Beijing, China; ^7^ School of Basic Medical Sciences, Henan University, Kaifeng, China

**Keywords:** breast cancer, DCE-MRI, axillary lymph node, metastasis, radiomics

## Abstract

**Objective:**

To investigate the value of predicting axillary lymph node (ALN) metastasis based on intratumoral and peritumoral dynamic contrast-enhanced MRI (DCE-MRI) radiomics and clinico-radiological characteristics in breast cancer.

**Methods:**

A total of 473 breast cancer patients who underwent preoperative DCE-MRI from Jan 2017 to Dec 2020 were enrolled. These patients were randomly divided into training (n=378) and testing sets (n=95) at 8:2 ratio. Intratumoral regions (ITRs) of interest were manually delineated, and peritumoral regions of 3 mm (3 mmPTRs) were automatically obtained by morphologically dilating the ITR. Radiomics features were extracted, and ALN metastasis-related radiomics features were selected by the Mann-Whitney *U* test, Z score normalization, variance thresholding, K-best algorithm and least absolute shrinkage and selection operator (LASSO) algorithm. Clinico-radiological risk factors were selected by logistic regression and were also used to construct predictive models combined with radiomics features. Then, 5 models were constructed, including ITR, 3 mmPTR, ITR+3 mmPTR, clinico-radiological and combined (ITR+3 mmPTR+ clinico-radiological) models. The performance of models was assessed by sensitivity, specificity, accuracy, F1 score and area under the curve (AUC) of receiver operating characteristic (ROC), calibration curves and decision curve analysis (DCA).

**Results:**

A total of 2264 radiomics features were extracted from each region of interest (ROI), 3 and 10 radiomics features were selected for the ITR and 3 mmPTR, respectively. 5 clinico-radiological risk factors were selected, including lesion size, human epidermal growth factor receptor 2 (HER2) expression, vascular cancer thrombus status, MR-reported ALN status, and time-signal intensity curve (TIC) type. In the testing set, the combined model showed the highest AUC (0.839), specificity (74.2%), accuracy (75.8%) and F1 Score (69.3%) among the 5 models. DCA showed that it had the greatest net clinical benefit compared to the other models.

**Conclusion:**

The intra- and peritumoral radiomics models based on DCE-MRI could be used to predict ALN metastasis in breast cancer, especially for the combined model with clinico-radiological characteristics showing promising clinical application value.

## 1 Introduction

Breast cancer has become the leading cause of cancer-related death among women worldwide (1). It has been reported that the 5-year survival rate for breast cancer patients with axillary lymph node (ALN) metastasis is 14% lower than that without metastasis (2). Accurate assessment of ALN status is critical for the clinical staging, selection of appropriate management and prognosis evaluation of breast cancer patients (3). Axillary lymph node dissection (ALND) remains the definitive treatment for palpable axillary positive patients, but this invasive procedure may result in postoperative complications. Sentinel lymph node biopsy (SLNB) has replaced ALND as the standard ALN assessment procedure for palpable axillary negative patients. However, it has a high false-negative rate of 7.8-27.3%, potentially causing challenges in subsequent treatment and management (4–7).

Imaging examinations, such as mammography, ultrasound, and MRI, are commonly used for preoperative assessment in ALN metastasis clinically. However, the results of these examinations can be subjectively affected by the experience of radiologists, which could lead to relatively elevated rates of missed diagnoses (8). Preoperative ultrasound-guided needle biopsy is also one of the clinically utilized methods for the assessment of ALN metastasis (9). However, it is difficult to reflect the whole heterogeneity due to the limited tissue samples obtained, and patients with negative biopsy results still need to undergo surgery to confirm the ALN pathological staging. Hence, the major problem is how to precisely evaluate the ALN with a noninvasive method.

Radiomics involves extracting quantitative features from medical images. Its goal is to explore connections between radiomics features and clinical information for better diagnosis and prognosis (10). DCE-MRI could provide crucial tumor hemodynamic information based on the multisequence imaging, and radiomics based on DCE-MRI has been corroborated by multiple studies for predicting ALN metastasis (11, 12). Peritumoral radiomics can reflect the microenvironment closely related to tumor growth and invasion (13), while research exploring their relevance to ALN metastasis remains limited. Additionally, clinico-radiological characteristics of breast cancer have been shown to be correlated with ALN metastasis (14, 15). This study aims to construct and validate preoperative predictive models for ALN metastasis based on both intra- and peritumoral DCE-MRI radiomics features and clinico-radiological characteristics in breast cancer.

## 2 Methods

### 2.1 Patient population

**Figure 1:**
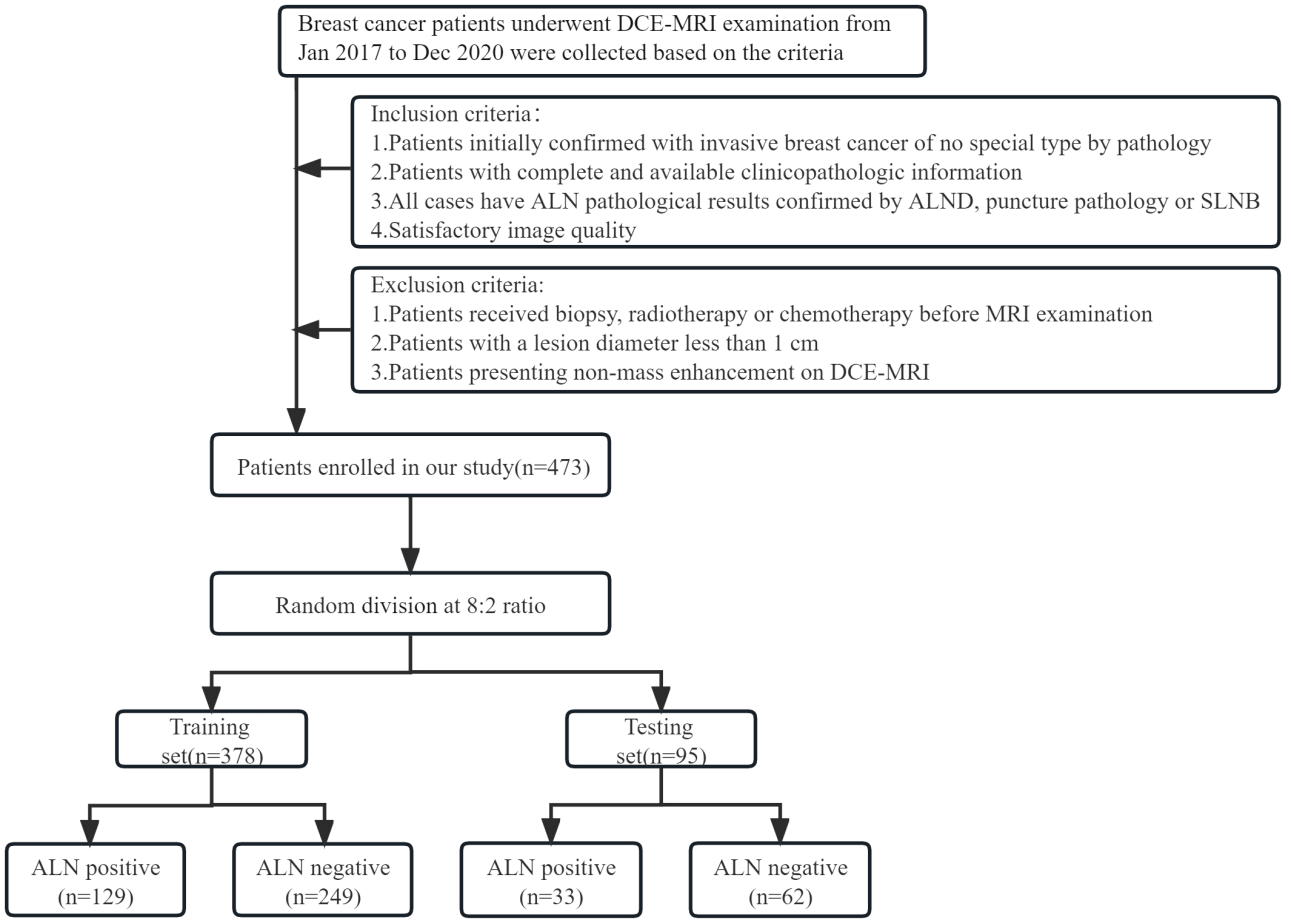
Flow chart for selection and grouping of populations according to inclusion and exclusion criteria.

The study was approved by the Ethics Committee of Henan Provincial People’s Hospital (No: 2022-124), and the participants informed consent requirement was waived. Breast cancer patients who underwent initial DCE-MRI examination in our hospital from Jan 2017 to Dec 2020 were retrospectively enrolled. The inclusion criteria were as follows: (1). Patients were initially confirmed with invasive breast cancer of nonspecific type (IBC-NST) by pathology; (2). Patients with complete and available clinicopathologic information; (3). All cases have ALN pathological results confirmed by ALND, puncture pathology or SLNB; (4). Image quality was satisfactory. The exclusion criteria were as follows: (1). Breast cancer patients who underwent biopsy, radiotherapy or chemotherapy before MRI examination; (2). Patients with a lesion diameter less than 1 cm; (3). Patients presenting non-mass enhancement on DCE-MRI (In consideration of the accuracy of ROI delineation, non-mass enhanced lesions were excluded because of their unclear boundaries and various distribution patterns). In this study, positive ALNs were determined by the results of ALND and puncture biopsy, while negative ALNs were mainly determined by the results of ALND and SLNB. Patients with negative SLNB were directly considered to have no ALN metastasis. According to this criterion, patients were divided into ALN-positive and ALN-negative groups. A total of 473 patients were enrolled based on the inclusion and exclusion criteria, including 162 positive and 311 negative ALN patients. Patients were randomly divided into training (n = 378, mean age 50.20 ± 10.21) and testing sets (n = 95, mean age 47.45 ± 9.15) at a ratio of 8:2. The study flow chart is shown in **Figure 1**.

### 2.2 DCE-MRI examination

Breast MRI examination was performed by 3.0T MR imaging devices and dedicated breast phased-array surface coils (GE Medical Systems Discovery MR750, Milwaukee). All enrolled patients lay in the prone position on the breast coil, with both breasts freely hanging pendulous. The main MRI sequence scanning parameters were as follows: unenhanced T1-weighted axial sequences (TR/TE=792/10 mms, FOV=340340 mm, matrix=512 512, slice thickness=5 mm, interval=2.5 mm, and number of slices=24). Dynamic contrast-enhanced scanning was performed using the T1-weighted volume imaging for breast assessment (VIBRANT) technique (TR/TE,3.8/1.6 mms; slice thickness, 1.1 mm; field of view, 751 340 mm; matrix scan, 512 512; phase,8). Intravenous access was established using a 12G intravenous indwelling needle before the examination. The contrast medium (Gado-linium-DTPA; Magnevist, Schering, Germany, 0.2 mmol/kg) was intravenously administered as a bolus injection to the patients undergoing contrast-enhanced MRI, followed by a 20 ml saline flush. A total of 8 phases were scanned, with 124 scanning layers for each phase.

### 2.3 Clinico-radiological characteristics

Clinical characteristics were obtained from the electronic medical records, including age, pathological grade, expression of estrogen receptor (ER), progesterone-receptor (PR), human epidermal growth factor receptor 2 (HER2), antigen Ki67, and vascular cancer thrombus status. ER expression was categorized into low (≤10%) and high (> 10%) groups. PR and Ki67 expression were classified as low (PR ≤ 20%, Ki67 <20%) and high (PR>20%, Ki67≥20%) groups based on a cut-off of 20% (16, 17). HER2 status was confirmed by immunohistochemistry (IHC) and fluorescence *in situ* hybridization (FISH). Radiological characteristics were analyzed by 2 radiologists with more than 5 years of experience in breast imaging. They were blinded to the pathological results, and a consensus decision was made in cases of discrepancy. All the original images acquired after scanning were transmitted to the AW4.6 postprocessing workstation. The ROIs were selected at the obvious lesion enhancement site, avoiding areas with hemorrhage, necrosis and calcification to analyze the time-signal intensity curve (TIC), which were classified into 3 classical types: type I – inflow type, type II – platform type, and type III – outflow type. The assessment was mainly based on the second edition of the Breast Imaging Reporting and Data System (BI-RADS) for breast MRI (18). Background parenchymal enhancement (BPE) type, lesion size (longest diameter), MR-reported ALN status and TIC type were recorded as the radiological characteristics.

### 2.4 Image segmentation

**Figure 2:**
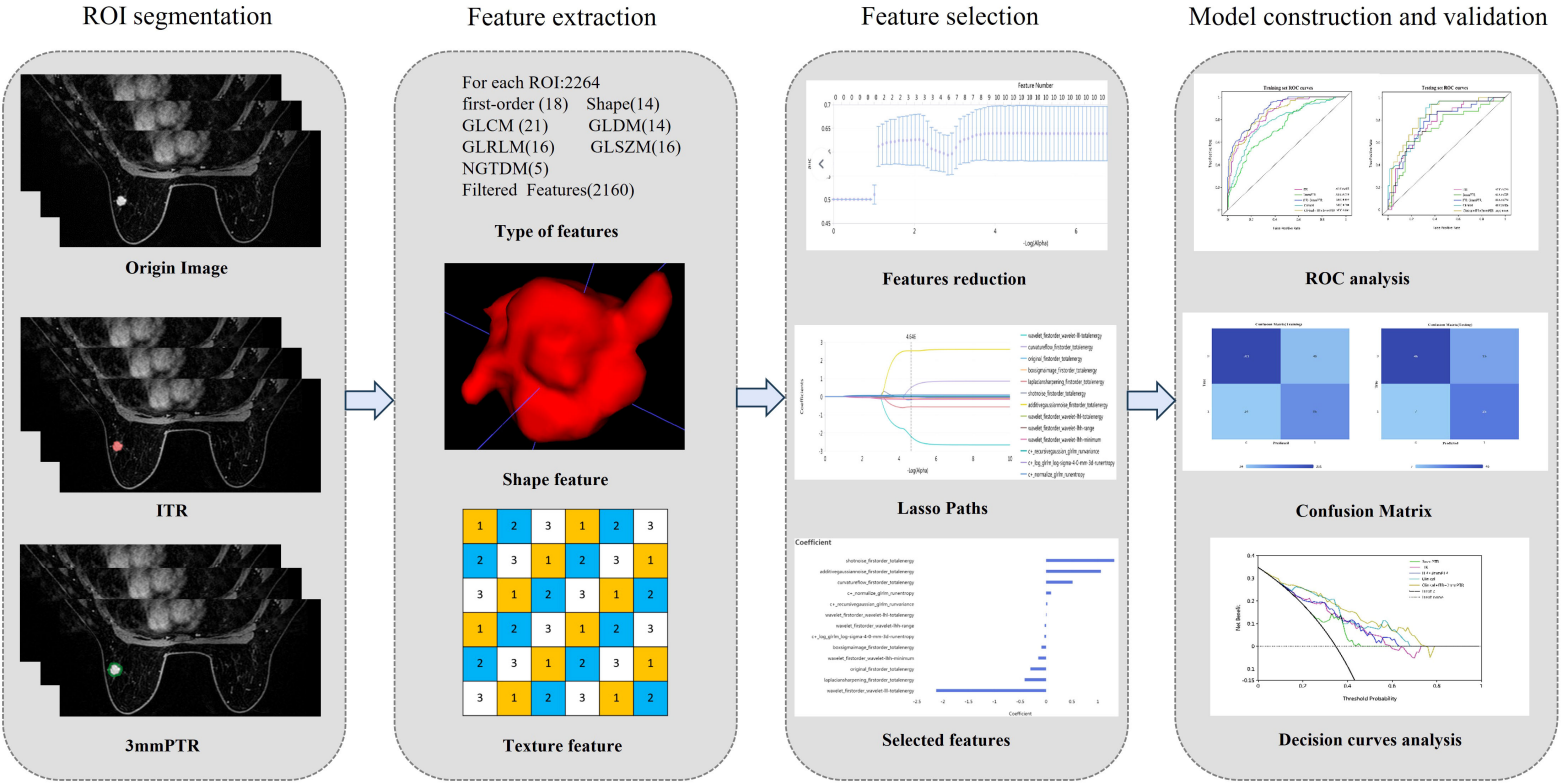
The workflow of radiomics. (1) Outlining ROI by manual joint automatic algorithm (2) Extraction of high-throughput features based on 2 different ROIs respectively (3) Feature selection (4) Model construction and validation. ITR: intratumoral region; 3mmPTR: peritumoral region of 3 mm.

**Figure 3:**
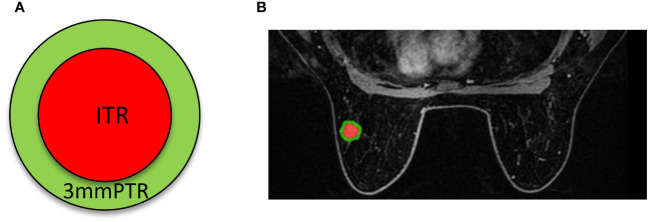
Schematics **(A)** and examples **(B)** of different ROI segmentation schemes. The red region represents the ITR, and the green region represents the 3 mm PTR. ITR: intratumoral region; 3mmPTR: peritumoral region of 3 mm.

The workflow of the radiomics analysis is illustrated in **Figure 2**. The third phase of DCE-MRI images, in which lesions achieved sufficient enhancement (19), were anonymously exported from the picture archiving and communication systems (PACS) and saved in DICOM format. Using ITK-SNAP software (Version 3.8.0, http://www.itk-snap.org), a breast radiologist with more than 5 years of experience manually delineated the intratumoral region (ITR) slice-by-slice, avoiding necrotic areas. The ITRs were verified by an associate chief physician with over 10 years of experience in breast radiology. The peritumoral region (PTR) was generated by morphologically dilating the ITR outwards by 3 mm using the uRP platform (uAI research portal, https://www.uii-ai.com/en/uai/scientifific-research), which is a clinical research platform integrating AI module algorithms (20). PTR portions extending beyond the breast parenchyma were removed manually. Each lesion obtained 2 ROIs, including ITR and 3 mm PTR (**Figure 3**).

### 2.5 Radiomics feature extraction and selection

A total of 2264 radiomics features were extracted from each ROI through the uRP platform. Feature selection was performed to avoid overfitting the models, and this process was conducted in the training set. Firstly, Mann-Whitney *U* test was performed to select ALN metastasis -related features. Subsequently, Z score normalization was used to reduce feature dimensionality differences, features with variance over threshold (0.1) were retained by variance threshold, and high p value features were filtered using K-Best (i.e., F value method). Finally, least absolute shrinkage and selection operator (LASSO) regression was applied to remove features with high collinearity, thereby obtaining the optimal feature subset based on the 2 ROIs. The detailed features and their respective coefficients are shown in the **Supplementary Data**.

### 2.6 Model construction and performance assessment

First, univariate analysis and binary logistic regression were used to select clinico-radiological independent factors of ALN metastasis. Second, the optimal radiomics features and clinico-radiological independent factors were standardized using preprocessing methods to unify dimensions respectively. Bagging decision tree was applied to construct predictive models. Detailed preprocessing methods are shown in the **Supplementary Data**. Finally, based on the model performance assessment indices, 3 optimal models were selected, including ITR, 3 mm PTR and clinico-radiological models. Then, 2 additional models were constructed based on the optimal radiomics features from intra- and peritumoral regions and clinico-radiological independent factors, including the ITR + 3 mm PTR model and the combined (ITR+3 mmPTR+ clinico-radiological) model.

The performance of the models was assessed by sensitivity, specificity, accuracy, F1 score and area under the curve (AUC) of receiver operating characteristic (ROC). The calibration of the models was assessed using the Hosmer-Lemeshow goodness-of-fit test and calibration curves. Decision curve analysis (DCA) compared the net clinical benefits of the models across a range of threshold probabilities.

### 2.7 Statistical analysis

Statistical analysis was performed using SPSS software (V.26.0) and R software (V. 4.3.1). Quantitative variables were compared using the *t* test or Mann-Whitney *U* test, while qualitative variables were compared using the χ2 test or Fisher’s test, if one of the theoretical frequencies was less than 1, the likelihood ratio chi-square was adopted. The ordered classified variables were compared with the rank sum test. Binary logistic regression analysis was performed to select clinico-radiological independent factors, which were used to construct predictive models combined with radiomics features. The AUCs of different models were compared by the DeLong test, The correction for multiple comparisons was conducted using Bonferroni correction. The value of sensitivity, specificity, accuracy, and F1Score were obtained based on the cut-off 0.5. P *<*0.05 was considered statistically significant.

## 3 Results

### 3.1 Clinico-radiological characteristics

**Figure 4:**
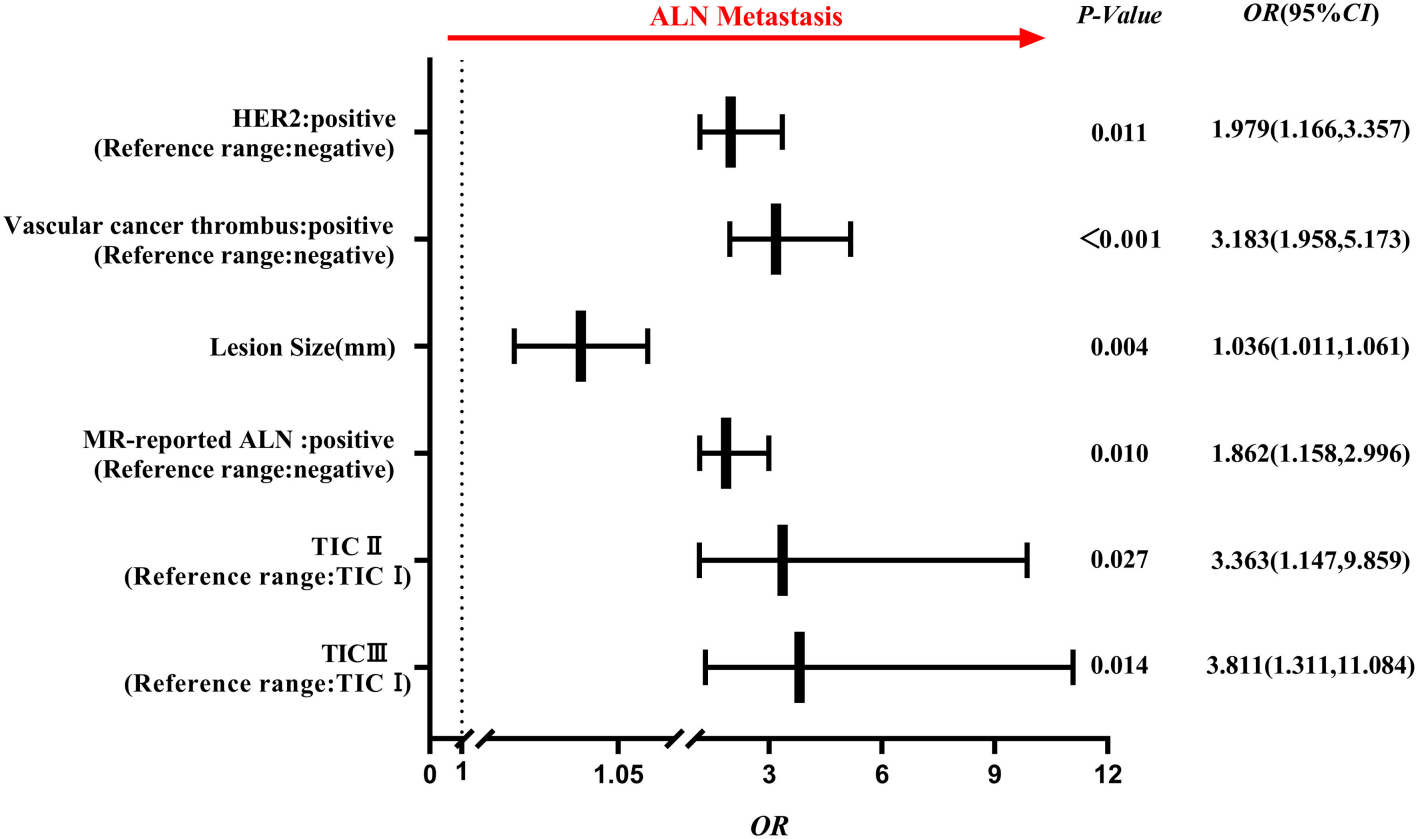
Forest plot of independent factors for predicting ALN metastasis based on clinico-radiological characteristics of breast cancer. The results revealed that positive HER2 expression, positive vascular cancer thrombus, lesion size, MR-reported positive ALN and TIC (II, III) type were independent risk factors for ALN metastasis.

Comparing the clinico-radiological characteristics between the training and testing sets, there were no significant differences except for age and MR-reported ALN status (both *p*<0.05, **Table 1**). In the ALN-positive group, the percentages of positive HER2 expression, positive vascular cancer thrombus, MR reported-positive ALN and type II~III TIC were higher than those in the negative group, lesion size was also larger in the positive group (all *p*<0.05, **Table 2**), and they were used to select independent risk factors for ALN metastasis by binary logistic regression. The results showed that positive HER2 expression (*OR* = 1.979, *P* = 0.011), positive vascular cancer thrombus (*OR* = 3.183, *P* < 0.001), larger lesion size (*OR* = 1.036, *P* = 0.004), MR-reported positive ALN (*OR* = 1.862, *P* = 0.010), and TIC types (II: *OR* = 3.363, *P* = 0.027; III: *OR* = 3.811, *P* = 0.014) were independent risk factors for ALN metastasis (**Figure 4**).

**Table 1:** Comparison of clinico-radiological characteristics between the training and testing sets.

Characteristics	Training set(n=378)	Testing set(n=95)	*p*
Age (year, $\bar{x}\pm \text{s}$ )	50.20±10.21	47.45±9.15	**0.017**
Pathological grade			0.257
Grade I	5 (1.3%)	3 (3.2%)	
Grade II	238 (63%)	63 (66.3%)	
Grade III	135 (35.7%)	29 (30.5%)	
ER expression			0.759
≤10%	112 (29.6%)	26 (27.4%)	
>10%	266 (70.4%)	69 (72.6%)	
PR expression			0.984
≤20%	200 (52.9%)	51 (53.7%)	
>20%	178 (47.1%)	44 (46.3%)	
HER2 expression			0.549
negative	289 (76.5%)	76 (80.0%)	
positive	89 (23.5%)	19 (20.0%)	
KI67 expression			0.073
<20%	95 (25.1%)	15 (15.8%)	
≥20%	283 (74.9%)	80 (84.2%)	
Vascular cancer thrombus			0.342
negative	257 (68.0%)	70 (73.7%)	
positive	121 (32.0%)	25 (26.3%)	
BPE type			0.552
No enhancement	66 (17.5%)	17 (17.9%)	
Mild enhancement	196 (51.9%)	53 (55.8%)	
Moderate enhancement	102 (27.0%)	21 (22.1%)	
Marked enhancement	14 (3.7%)	4 (4.2%)	
Lesion Size [mm/M(Q1,Q3)]	20 (16, 28)	22 (15, 28)	0.828
MR reported- ALN			**0.018**
negative	236 (62.4%)	46 (48.4%)	
positive	142 (37.6%)	49 (51.6%)	
TIC type			0.241
I	34 (9.0%)	4 (4.2%)	
II	150 (39.7%)	36 (37.9%)	
III	194 (51.3%)	55 (57.9%)	

The bold values presented indicate statistically significant p-values.

**Table 2:** Comparison of clinico-radiological characteristics between different ALN groups in the training and testing sets.

Characteristics	Training set(n=378)			Testing set(n=95)		
	Positive (n=129)	negative (n=249)	*p*	positive (n=33)	negative (n=62)	*p*
Age (year, $\bar{x}\pm \text{s}$ )	49.91±10.57	50.35±10.03	0.698	46.58±9.24	47.92±9.15	0.499
Pathological grade			0.088			0.244
Grade I	2(1.6%)	3(1.2%)		0(0.0%)	3(4.8%)	
Grade II	73(56.6%)	165(66.3%)		21(63.6%)	42(67.7%)	
Grade III	54(41.9%)	81(32.5%)		12(36.4%)	17(27.4%)	
ER expression			0.170			0.640
≤10%	44(34.1%)	68(27.3%)		10(30.3%)	16(25.8%)	
>10%	85(65.9%)	181(72.7%)		23(69.7%)	46(74.2%)	
PR expression			0.143			0.156
≤20%	75(58.1%)	125(50.2%)		21(63.6%)	30(48.4%)	
>20%	54(41.9%)	124(49.8%)		12(36.4%)	32(51.6%)	
HER2 expression			**0.003**			**0.018**
negative	87(67.4%)	202(81.1%)		22(66.7%)	54(87.1%)	
positive	42(32.6%)	47(18.9%)		11(33.3%)	8(12.9%)	
KI67 expression			0.063			0.191
<20%	25(19.4%)	70(28.1%)		3(9.1%)	12(19.4%)	
≥20%	104(80.6%)	179(71.9%)		30(90.9%)	50(80.6%)	
Vascular cancer thrombus			**<0.001**			**<0.001**
negative	64(49.6%)	193(77.5%)		17(51.5%)	53(85.5%)	
positive	65(50.4%)	56(22.5%)		16(48.5%)	9(14.5%)	
BPE type			0.340			0.540
No enhancement	19(14.7%)	47(18.9%)		6(18.2%)	11(17.7%)	
Mild enhancement	75(58.1%)	121(48.6%)		20(60.6%)	33(53.2%)	
Moderate enhancement	30(23.3%)	72(28.9%)		6(18.2%)	15(24.2%)	
Marked enhancement	5(3.9%)	9(3.6%)		1(3.0%)	3(4.8%)	
Lesion Size [mm/M(Q1,Q3)]	24.0 (18.5,31.0)	19.0 (15.0,25.0)	**<0.001**	27.0 (22.5,33.0)	17.0 (14.8,25.0)	**<0.001**
MR reported- ALN			**<0.001**			**<0.001**
negative	64(49.6%)	172(69.1%)		4(12.1%)	42(67.7%)	
positive	65(50.4%)	77(30.9%)		29(87.9%)	20(32.3%)	
TIC type			**0.034**			0.043*
I	5(3.9%)	29(11.6%)		0(0.0%)	6(9.7%)	
II	51(39.5%)	99(39.8%)		13(39.4%)	28(45.2%)	
III	73(56.6%)	121(48.6%)		20(60.6%)	28(45.2%)	

*One of the theoretical frequencies was less than 1, the likelihood ratio chi-square result was adopted here. The bold values presented indicate statistically significant p-values.

### 3.2 Feature extraction and selection

A total of 2264 radiomics features were extracted from each ROI, including 18 first-order statistical features, 14 shape features, 72 texture features and 2160 filtered features (i.e., high-order statistical features). 3 and 10 features were finally selected as the optimal features based on ITR and 3 mm PTR using the LASSO method. The ITR contained 3 high-order statistical features, while the 3 mm PTR contained 1 first-order statistical feature and 9 high-order statistical features (20).

### 3.3 Construction and validation of models

**Figure 5:**
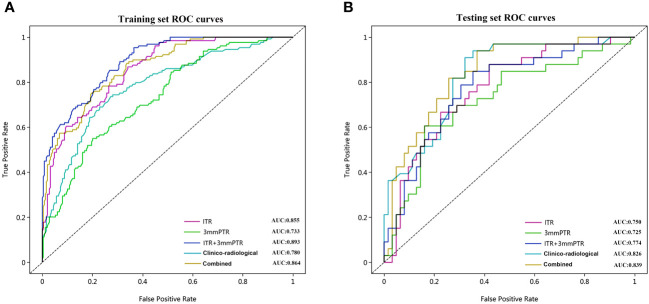
The ROC curves of the 5 models used to predict ALN metastasis in breast cancer patients in the training **(A)** and testing **(B)** sets. ROC, receiver operating characteristic; AUC, area under the curve of ROC; Combined, means the ITR+3 mmPTR+ clinico-radiological model.

The performances of the models based on ITR, PTR and clinico-radiological characteristics are shown in **Table 3**. The AUC of the combined model (ITR+3 mmPTR+clinico-radiological) in the testing set was 0.839, which was the highest compared to the other 4 models (ITR, 3 mm PTR, ITR + 3 mm PTR, clinico-radiological, **Figure 5**). The combined model also achieved the highest specificity (74.2%), accuracy (75.8%) and F1 Score (69.3%). The DeLong test showed that the AUCs of the 3 mm PTR and ITR models were significantly different from that of the combined model in the testing set (*P*=0.047 and 0.036, respectively), and the results are shown in **Table 4**.

**Table 3:** Performances of the models based on radiomics features and clinico-radiological characteristics.

Models/Groups	Sensitivity	Specificity	Accuracy	F1Score	AUC (95% CI)
ITR					
Training	66.7%	81.5%	76.5%	65.9%	0.855 (0.818,0.893)
Testing	60.6%	71.0%	67.4%	56.3%	0.750 (0.649,0.850)
3mmPTR					
Training	61.2%	71.9%	68.3%	56.8%	0.733 (0.682,0.785)
Testing	66.7%	71.0%	69.5%	60.3%	0.725 (0.614,0.836)
ITR + 3mmPTR					
Training	77.5%	78.3%	78.0%	70.7%	0.893 (0.862,0.924)
Testing	72.7%	72.6%	72.6%	64.9%	0.774 (0.675,0.872)
Clinico-radiological					
Training	75.2%	68.7%	70.9%	63.8%	0.780 (0.731,0.830)
Testing	81.8%	69.4%	73.7%	68.4%	0.826 (0.741,0.911)
Combined^#^					
Training	73.6%	80.7%	78.3%	69.9%	0.864 (0.827,0.901)
Testing	78.8%	74.2%	75.8%	69.3%	0.839 (0.758,0.920)

#Represents the combined model of ITR + 3mmPTR+ clinico-radiological.

**Table 4:** Significant level of Delong test between the combined model and the other models.

Groups	3mmPTR	ITR	ITR + 3mmPTR	Clinico-radiological
Training	**<0.001**	0.681	0.106	**<0.001**
Testing	**0.047**	**0.036**	0.142	0.649

The combined model represents ITR + 3mmPTR+ clinico-radiological model. The bold values presented indicate statistically significant p-values.

**Figure 6:**
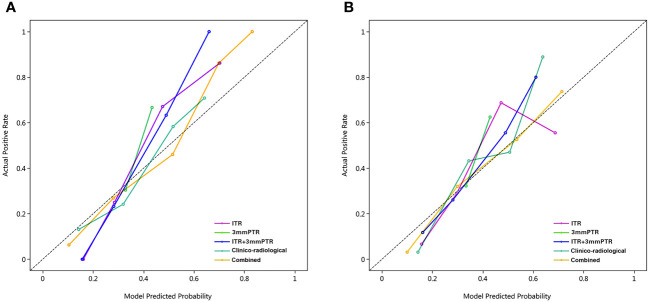
Calibration curves of the 5 models in the training and testing sets. The calibration curves show the agreement between the predicted probability of ALN metastasis and the actual metastasis outcomes. The y-axis represents the actual metastasis rate. The x-axis represents the predicted metastasis probability. The diagonal line represents ideal prediction. **(A)** Training set; **(B)** Testing set; Combined, means the ITR+3 mmPTR+ clinico-radiological model.

**Figure 7:**
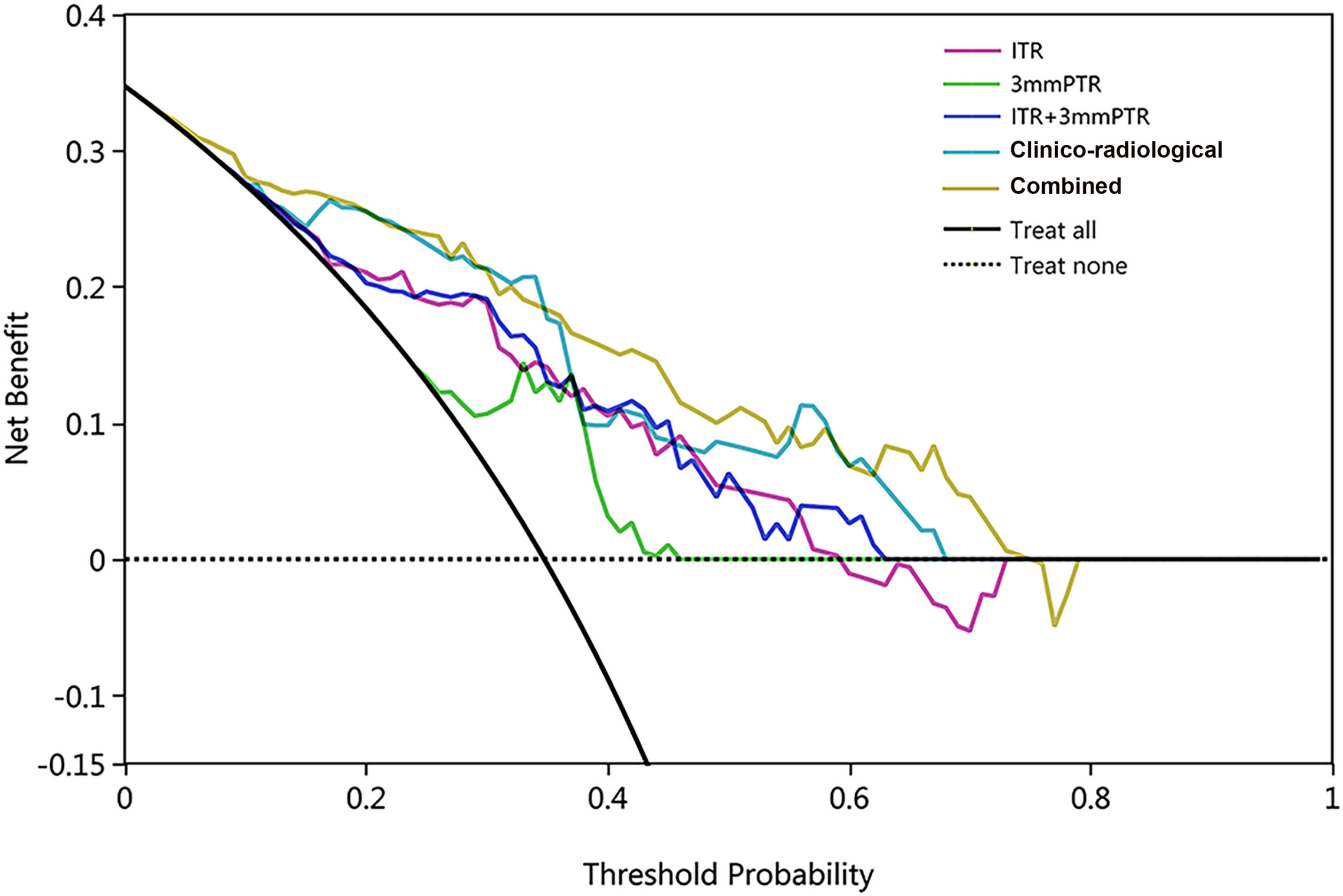
The DCA of 5 models in the testing set, with threshold probability on the x-axis and net benefit on the y-axis. The black solid and dashed lines represent the ‘treat-all’ and ‘treat-none’ strategies. The combined model (ITR+3mmPTR+clinico-radiological) showed the highest net benefit within the threshold probability range of 0.04-0.76, with the widest applicable range.

The calibration curves demonstrated good consistency between predicted risks and observed probabilities across the 5 datasets. (**Figure 6**). The DCA showed that the combined model had the best clinical net benefit across threshold probabilities of 0.04-0.76 and widest applicable range compared to other models (**Figure 7**).

## 4 Discussion

As an essential prognostic factor of breast cancer, ALN status is critical for therapy decision- making. However, accurate preoperative assessment of ALN metastasis remains challenging for radiologists relying on medical imaging, for the high false-negative rate of morphological features on images. Radiomics is a method of data mining to extract crucial information from images, enabling tumor diagnosis, prognosis prediction, and other clinical applications (21). DCE-MRI radiomics has been reported to be useful for assessing ALN metastasis (22–24). Additionally, peritumoral radiomics can capture the heterogeneity of the tumor microenvironment, but few studies have utilized it to predict ALN metastasis. In this study, we constructed and validated a radiomics model based on features extracted from intratumoral and peritumoral regions, and the capability of the model for predicting ALN metastasis is impressive. Integrated with clinico-radiological characteristics, the combined model showed excellent performance in predicting ALN metastasis, exhibiting greater net benefits, which to some extent reduced unnecessary pathological surgeries.

Our results confirmed that previously reported clinico-radiological predictors, such as HER2 expression, lesion size and MR-reported ALN status, could be related to ALN metastasis (25, 26). The results showed that positive vascular tumor thrombus was a moderate intensity risk factor (*OR* = 3.183), which could be explained by the widespread lymphatic vessels in the breast. It make it easy for cancer cells draining to the ALN through lymphatic vessels infiltrating the breast lobules (27). Interestingly, type II and III TICs were also strongly correlated with ALN metastasis (II: OR = 3.363, III: OR = 3.811), although they were primarily used to differentiate benign and malignant breast lesions. And MR-reported ALN status can also contributes to enhance the predictive performance of the radiomics model in this study (28). In the testing set, the clinico-radiological model outperformed the radiomics models with an AUC of 0.826. However, it performed slightly inferior in the training set, which was probably caused by the instability of the characteristics or imbalanced grouping. However, when the ITR+3 mmPTR model was integrated with clinico-radiological characteristics, the AUC improved from 0.774 to 0.839 in the testing set. Therefore, clinico-radiological characteristics were valuable for assessing ALN metastasis. For the selected features in our study, we found the subclass of the optimal high-order statistical features were first-order statistics and gray level run length matrix (GLRLM) that belongs to a type of textural features. The first-order statistics features, such as the mean and median, reflect the distribution of voxel intensity for images. The results of our study are consistent with Yan et al (29), they demonstrated the first-order features were related to LN metastasis in endometrial cancer. The GLRLM features, commonly utilized to quantify regional heterogeneity differences, have been validated for their predictive value in ALN metastasis (30), consistent with our study findings.

Intratumoral radiomics was one of the most important components in predicting ALN metastasis. Notably, regarding the choice of the DCE phase, there is no consensus in defining which phase of the radiomics offers the best prediction performance. We chose the third phase of post-contrast DCE-MRI for radiomics analysis because it offers sufficient tumor enhancement, facilitating clear visualization of lesion boundaries and providing valuable hemodynamic and heterogeneity information (31), and the radiomics model based on the third phase exhibited good performance in this study. Chai et al (23). constructed a radiomics model based on the second phase of postcontrast imaging, obtaining an AUC of 0.850. Liu et al. (31)used radiomics features of the obvious enhancement phase according to the TIC, but the predictive performance was similar to that of Chai et al. Consequently, the selection of the optimal DCE phase needs further exploration.

Previous studies in predicting ALN metastasis have focused solely on the primary tumor but have seldom taken peritumoral radiomics into account for analysis (32, 33). Peritumoral radiomics is related to the proliferation and metastasis of malignant cells, infiltration lymphocytes, angiogenesis, and stromal reactions (34, 35). Studies have shown that models incorporating peritumoral radiomics can better identify the molecular subtypes of breast cancer and evaluate neoadjuvant chemotherapy efficacy (36, 37). In this study, we analyzed the potential radiomics features of both the primary tumor and peritumoral region for the assessment of ALN metastasis. Our results demonstrated that peritumoral radiomics had good performance in the training (AUC: 0.733) and testing sets (AUC: 0.725). When combined with intratumoral features, the AUC reached 0.893 (training set) and 0.774 (testing set). Although the AUC did not improve much in the testing set, the ITR + 3 mmPTR model showed improved sensitivity and accuracy by 12.1% and 5.2%, respectively, compared to the ITR model. The improved sensitivity helped avoid missing ALN metastasis to some extent, underscoring the optimization of the model through peritumoral radiomics integration. Though the size selection of peritumoral region is inconclusive currently, it is reported the performance of radiomics model will reduce when expanding peritumoral regions from 5 mm to 10 mm (38, 39). Zhou et al. also found higher accuracy with the proximal peritumoral region compared to the larger region (13).This maybe have some relationship with the localization between the tumor and peritumoral tissue. Our study assessed a 3 mm proximal peritumoral radiomics model with the AUC value of 0.725, which performed better than previous studies (38). Comparisons of radiomics models with different sizes of peritumoral regions were not conducted but will be explored using different sequences in the future.

The combined model was established by incorporating intra- and peritumoral radiomics features with clinico-radiological risk factors in our study, which showed higher predictive efficacy than that of the independent model of radiomics and clinico-radiological characteristics. The combined model achieved the highest AUC (0.839), specificity (74.2%), accuracy (75.8%) and F1 Score (69.3%) among the 5 models in the testing set. DCA also showed that the combined model had the best clinical net benefit and widest applicable range compared to the other models, which indicates that the combined model has promising clinical application value.

There are some strengths in our study. We performed radiomics feature selection using various preprocessing methods, an approach that often yields models with stable performance. Additionally, the larger sample size in the present study compared to most previous studies may enhance the reliability of the results. However, there are also some limitations. First, as a single-center retrospective study, there may be selection bias. And the results need external validation, which would be conducted using datasets from multiple institutions in the subsequent studies. Second, all pathological types were IBC-NST and relatively homogeneous, and future studies could expand to more diverse cancer types. Finally, the peritumoral region was obtained by dilating the tumor 3 mm in this study, but whether this is the optimal peritumoral region requires further refinement of the expanded range and validation.

## 5 Conclusion

In summary, peritumoral radiomics based on DCE-MRI is helpful for accurately predicting ALN metastasis preoperatively. The combined model utilizing clinico-radiological, intratumoral and peritumoral information has higher predictive performance than each individual approach. It is expected to be a reliable tool for predicting ALN metastasis in clinical practice.

## Data availability statement

The raw data supporting the conclusions of this article will be made available by the authors, without undue reservation.

## Ethics statement

The studies involving humans were approved by Ethics Committee of Henan Provincial People’s Hospital (No: 2022-124). The studies were conducted in accordance with the local legislation and institutional requirements. Due to the retrospective nature of this study, the requirement for informed consent from patients was waived.

## Author contributions

YXW: Conceptualization, Data curation, Formal Analysis, Investigation, Methodology, Software, Validation, Visualization, Writing – original draft. YS: Data curation, Investigation, Writing – original draft. YXG: Data curation, Investigation, Writing – original draft. MH: Writing – original draft. YG: Writing – original draft. QW: Software, Writing – review & editing. SL: Writing – original draft. JL: Writing – original draft. XS: Funding acquisition, Writing – review & editing. YPW: Software, Writing – review & editing. MW: Conceptualization, Writing – review & editing. HT: Conceptualization, Funding acquisition, Project administration, Supervision, Writing – review & editing.
