# Supplementary material for: Clinical study on the prediction of ALN metastasis based on intratumoral and peritumoral DCE-MRI radiomics and clinico-radiological characteristics in breast cancer
Source: Front Oncol. 2024 Mar 19;14:1357145. doi: 10.3389/fonc.2024.1357145 (PMC10985134; doi:10.3389/fonc.2024.1357145)
Supplement: Supplementary file 1 [file DataSheet_1.docx]

***Supplementary Material***

***Radiomics feature extraction***

The feature extraction was performed by the in-house algorithm of the uRP platform (uAI research portal, <https://www.uii-ai.com/en/uai/scientifific-research>). A total of 2264 radiomics features were automatically extracted from each obtained ROI, including 104 original radiomics features and 2160 filtered features.

The 104 original radiomics features included 18 histogram features that reflect the distribution of voxel intensity in the image, 14 shape features that provide quantitative geometric properties of the ROI, and 72 texture features include 16 gray level size zone matrix (GLSZM) features, 21 gray level co-occurrence matrix (GLCM) features, 5 neighboring gray-tone difference matrix (NGTDM) features, 16 gray level run length matrix (GLRLM) features, and 14 gray levels dependent matrix (GLDM) features, which reflect intensity relationships between voxels within the image.

The derived features were mainly obtained by applying 24 fifilters (box mean, additive Gaussian noise, binomial blur, curvature flow, box-sigma, normalize, Laplacian sharpening, discrete Gaussian, mean, speckle noise, recursive Gaussian, shot noise, LoG (sigma: 0.5, 1, 1.5, 2), and wavelets (LLL, LLH, LHL, LHH, HLL, HLH, HHL, HHH)). For each filter, features are computed for 7 categories, including histogram, GLCM, GLRLM, GLSZM, NGTDM, GLDM, and shape. 2160 filtered features were finally obtained, which reflect the repetition patterns, edges and histogram gradients within the images. The detailed features and filters are illustrated in Table S1 and Table S2.

Table S1 2,264 radiomics features generated from one specific image

| Filter  Category | Histogram  (n=450) | GLCM  (n=525) | GLRLM  (n=350) | GLSZM  (n=400) | NGTDM  (n=125) | GLDM  (n=400) | Shape  (n=14) |
| --- | --- | --- | --- | --- | --- | --- | --- |
| Original image | 18 | 21 | 16 | 16 | 5 | 14 | 14 |
| Shot noise | 18 | 21 | 16 | 16 | 5 | 14 | - |
| Speckle noise | 18 | 21 | 16 | 16 | 5 | 14 | - |
| Additive Gaussian noise | 18 | 21 | 16 | 16 | 5 | 14 | - |
| Binomial blur image | 18 | 21 | 16 | 16 | 5 | 14 | - |
| Mean filter | 18 | 21 | 16 | 16 | 5 | 14 | - |
| Normalize filter | 18 | 21 | 16 | 16 | 5 | 14 | - |
| Box mean filter | 18 | 21 | 16 | 16 | 5 | 14 | - |
| Box signal image | 18 | 21 | 16 | 16 | 5 | 14 | - |
| Recursive Gaussian | 18 | 21 | 16 | 16 | 5 | 14 | - |
| Discrete Gaussian | 18 | 21 | 16 | 16 | 5 | 14 | - |
| *Wavelet (8) | 144 | 168 | 128 | 128 | 40 | 112 | - |
| *Laplacian of Gaussian (4) | 72 | 84 | 64 | 64 | 20 | 56 | - |
| Laplacian sharpening | 18 | 21 | 16 | 16 | 5 | 14 | - |
| Curvature flow | 18 | 21 | 16 | 16 | 5 | 14 | - |

* Wavelet filter owns 8 cases (i.e., HHH, HHL, HLH, LHH, HLL, LHL, LLH, LLL) and Laplacian of Gaussian filter owns 4 cases (i.e., sigma 0.5 mm, 1.0 mm, 1.5 mm, 2.0 mm).

Table S2 Names of different types of radiomics features and filters

| Category (Quantity) | Radiomics Features |
| --- | --- |
| Filter (n_f=14) | BoxMean, AdditiveGaussinNoise, BinomialBlurImage, CurvatureFlow, BoxsigmaImage, LoG, Wavelet, Normalize, LaplacianSharpening, DiscreteGaussian, Mean, SpeckleNoise, RecursiveGaussian, ShotNoise |
| Shape-based (n=14) | Area, Perimeter, Sphericity, Elongation, Extent, Circularity, Solidity, Eccentricity, Equivalent diameter, Major axis length, Minor axis length, Perimeter to area ratio, Maximum 2D diameter, Spherical disproportion |
| First Order Statistics / Histogram (n=450) | Energy, Total Energy, Entropy, Minimum, 10th percentile, 90th percentile, Maximum, Mean, Median, Interquartile Range, Range, Mean Absolute Deviation (MAD), Robust Mean Absolute Deviation (rMAD), Root Mean Squared (RMS), Standard Deviation, Skewness, Kurtosis, Variance, Uniformity |
| Gray Level Cooccurence Matrix (GLCM) (n=525) | Autocorrelation, Joint Average, Cluster Prominence, Cluster Shade, Cluster Tendency, Contrast, Correlation, Difference Average, Difference Entropy, Difference Variance, Joint Energy, Joint Entropy, Informational Measure of Correlation (IMC)(1~2), Inverse Difference Moment (IDM), Maximal Correlation Coefficient (MCC), Inverse Difference Moment Normalized (IDMN), Inverse Difference (ID), Inverse Difference Normalized (IDN), Inverse Variance, Maximum Probability, Sum Average, Sum Entropy, Sum of Squares |
| Gray Level Run Length Matrix (GLRLM) (n=350) | Short Run Emphasis (SRE), Long Run Emphasis (LRE), Gray Level Non-Uniformity (GLN), Gray Level Non-Uniformity Normalized (GLNN), Run Length Non-Uniformity (RLN), Run Length Non-Uniformity Normalized (RLNN), Run Percentage (RP), Gray Level Variance (GLV), Run Variance (RV), Run Entropy (RE), Low Gray Level Run Emphasis (LGLRE), High Gray Level Run Emphasis (HGLRE), Short Run Low Gray Level Emphasis (SRLGLE), Short Run High Gray Level Emphasis (SRHGLE), Long Run Low Gray Level Emphasis (LRLGLE), Long Run High Gray Level Emphasis (LRHGLE) |
| Gray Level Size Zone Matrix (GLSZM) (n=400) | Small Area Emphasis (SAE), Large Area Emphasis (LAE), Gray Level Non-Uniformity (GLN), Gray Level Non-Uniformity Normalized (GLNN), Size-Zone Non-Uniformity (SZN), Size-Zone Non-Uniformity Normalized (SZNN), Zone Percentage (ZP), Gray Level Variance (GLV), Zone Variance (ZV), Zone Entropy (ZE), Low Gray Level Zone Emphasis (LGLZE), High Gray Level Zone Emphasis (HGLZE), Small Area Low Gray Level Emphasis (SALGLE), Small Area High Gray Level Emphasis (SAHGLE), Large Area Low Gray Level Emphasis (LALGLE), Large Area High Gray Level Emphasis (LAHGLE) |
| Neighbouring Gray Tone Difference Matrix (NGTDM) (n=125) | Coarseness, Contrast, Busyness, Complexity, Strength |
| Gray Level Dependence Matrix (GLDM) (n=400) | Small Dependence Emphasis (SDE), Large Dependence Emphasis (LDE), Gray Level Non-Uniformity (GLN), Dependence Non-Uniformity (DN), Dependence Non-Uniformity Normalized (DNN), Gray Level Variance (GLV), Dependence Variance (DV), Dependence Entropy (DE), Low Gray Level Emphasis (LGLE), High Gray Level Emphasis (HGLE), Small Dependence Low Gray Level Emphasis (SDLGLE), Small Dependence High Gray Level Emphasis (SDHGLE), Large Dependence Low Gray Level Emphasis (LDLGLE), Large Dependence High Gray Level Emphasis (LDHGLE) |

***Radiomics features selection***

Feature selection was performed to mitigate high dimensionality, which could lead to computational inefficiency and overfitting. Mann Whitney U-test was firstly used to select the ALN metastasis-related radiomics features; Z-score normalization was used to unify the dimensions of the features. Subsequently, variance threshold and K-Best (i.e., F-value method) were utilized to filter the features, 10 features were preliminarily obtained. The least absolute shrinkage and selection operator (LASSO) regression was applied to select valuable radiomics features with nonzero coefficients. Finally, 3 types of high-order statistical features from ITR, 1 first-order statistical feature and 9 high-order statistical features from 3 mm PTR were selected as the optimal features. The detailed process of feature selection is illustrated in the Figure S1; The selected features and their respective coefficients are shown in Table S3 and Figure S2.


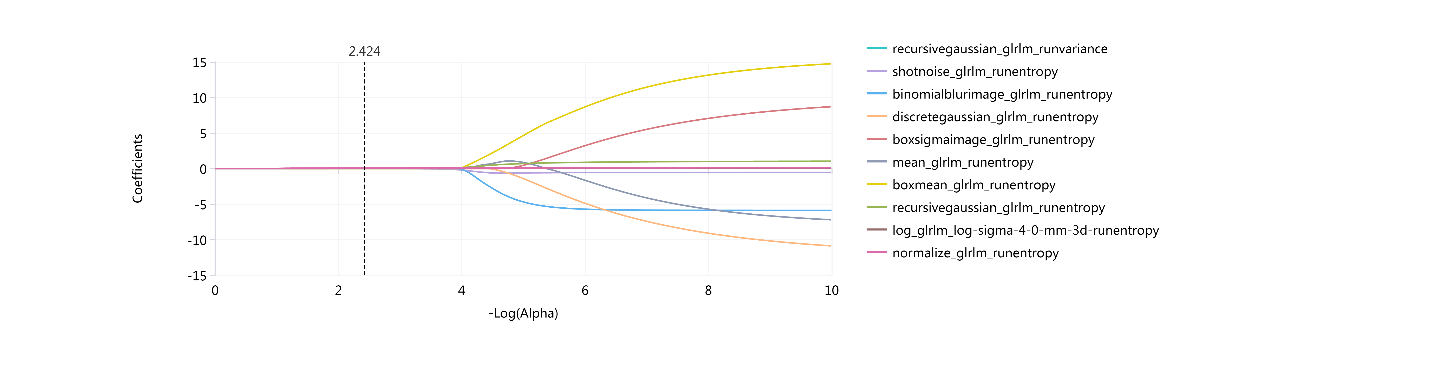


**A**


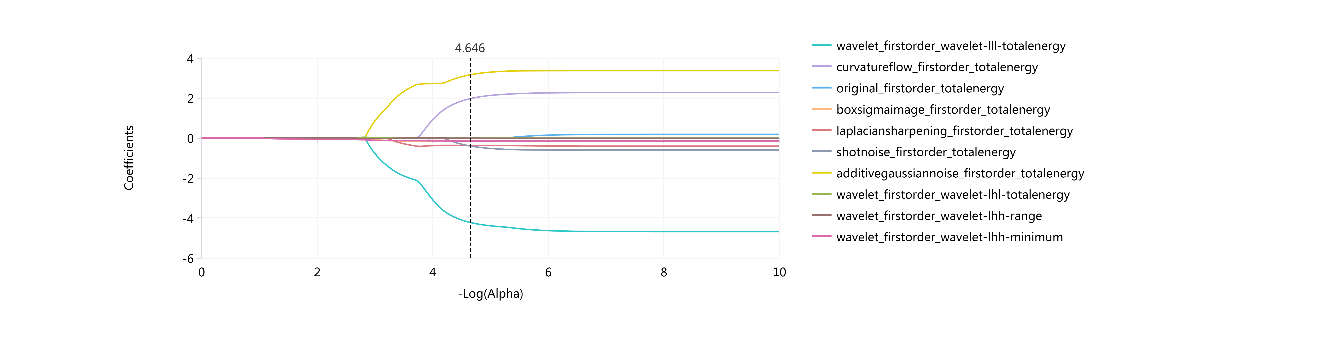


**B**

Figure S1. Lasso path depicting the feature selection process. Vertical lines indicate optimal λ values, resulting in 3 optimal features from ITR(**A**) and 10 optimal features from 3mm PTR(**B**).

Table S3 Optimal radiomics features related with ALN metastasis

| **Radiomics Feature name** | **Subclass** | **Filter** | **coefficient** |
| --- | --- | --- | --- |
| *normalize_glrlm_runentropy | GLRLM | Normalize | 0.073 |
| *recursivegaussian_glrlm_runvariance | GLRLM | RecursiveGaussian | 0.040 |
| *log_glrlm_log-sigma-4-0-mm-3d-runentropy | GLRLM | Laplacian of Gaussian | 0.005 |
| ^#^additivegaussiannoise_firstorder_totalenergy | First Order Statistics | AdditiveGaussinNoise | 1.656 |
| ^#^curvatureflow_firstorder_totalenergy | First Order Statistics | CurvatureFlow | 1.244 |
| ^#^shotnoise_firstorder_totalenergy | First Order Statistics | ShotNoise | 1.119 |
| ^#^wavelet_firstorder_wavelet-lhl-totalenergy | First Order Statistics | Wavelet | -0.002 |
| ^#^wavelet_firstorder_wavelet-lhh-range | First Order Statistics | Wavelet | -0.008 |
| ^#^boxsigmaimage_firstorder_totalenergy | First Order Statistics | BoxsigmaImage | -0.136 |
| ^#^wavelet_firstorder_wavelet-lhh-minimum | First Order Statistics | Wavelet | -0.154 |
| ^#^laplaciansharpening_firstorder_totalenergy | First Order Statistics | LaplacianSharpening | -0.173 |
| ^#^original_firstorder_totalenergy | First Order Statistics | original | -0.735 |
| ^#^wavelet_firstorder_wavelet-lll-totalenergy | First Order Statistics | Wavelet | -2.991 |

*Means optimal features extracted from ITR, ^#^ Means optimal features extracted from 3mm PTR


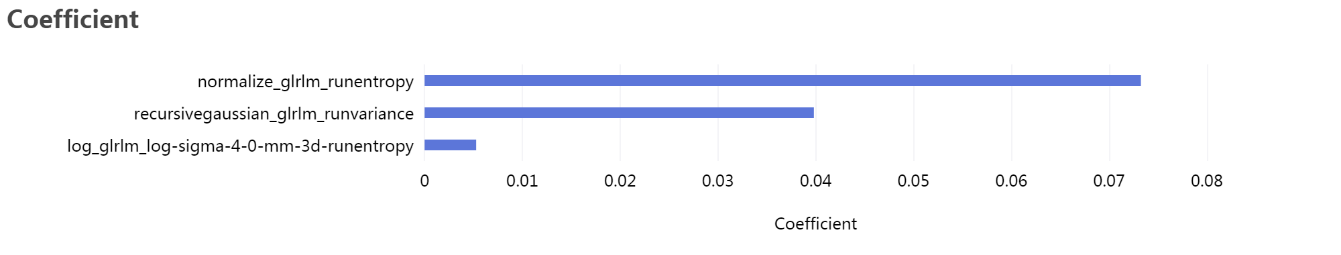


**B**

**A**


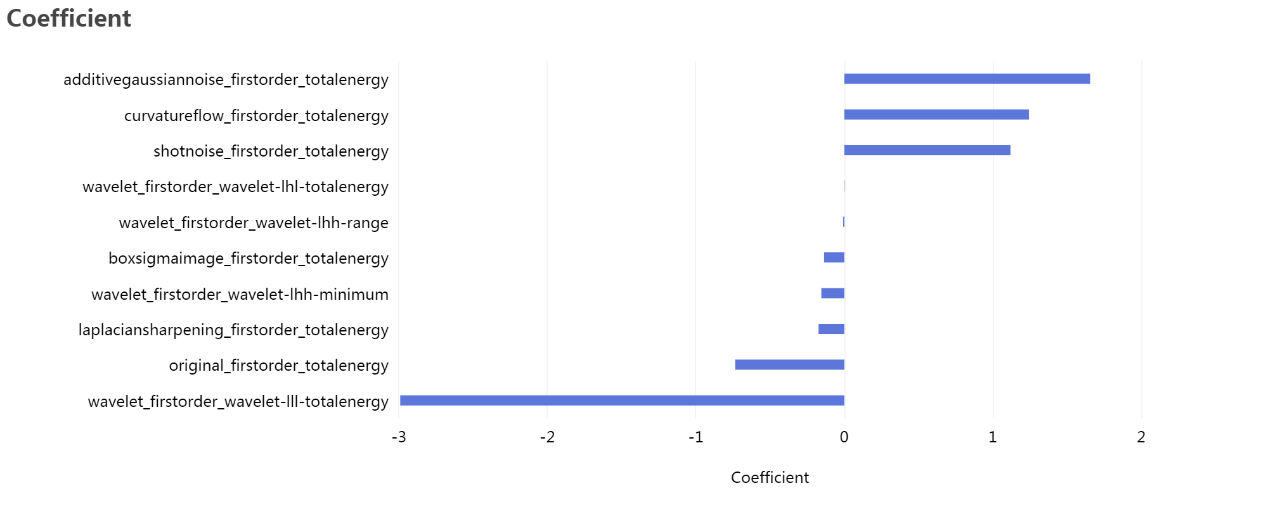


Figure S2. The correlation coefficients of the selected optimal features based on ITR (**A**)and 3mm PTR **(B)**.

***Construction of Prediction Models for ALN Metastasis***

We standardized the optimal radiomics features and clinico-radiological independent factors using 8 preprocessing methods to unify dimensions. Bagging decision tree were then applied to construct predictive models. The above 8 preprocessing methods and Bagging decision tree were carried out in a classification combination manner. Additionally, the 8 preprocessing methods include maximum minimum normalization, yeojohnson_transformer, L1_normalization, boxcox_transformer, absolute maximal normalization, Z-score normalization, L2_normalization, and quantitle_transformer. Finally, 3 optimal models were selected based on the performance assessment indexes, including ‘ITR, 3 mm PTR and clinico-radiological models’. Then, the optimal radiomics features extracted from intra- and peritumoral regions and clinico-radiological independent factors were combined, following the above model construction methodology, 2 models were constructed, including ‘ITR + 3 mm PTR, combined (ITR+3mmPTR+clinico-radiological) models’. The performance of these models was assessed by the sensitivity, specificity, accuracy, F1score and the area under the curve (AUC) of receiver operating characteristic (ROC).
